# The Infrapatellar Fat Pad in Osteoarthritis: From Pathophysiology to a Novel Therapeutic Target

**DOI:** 10.3390/ijms27052369

**Published:** 2026-03-03

**Authors:** Qianshuo Wang, Dingge Liu, Fan Hu, Langran Wang, Zhihua Zhang, Yuhao Yan, Xin Zhang

**Affiliations:** Beijing Key Laboratory of Sports Injuries, Institute of Sports Medicine, Peking University Third Hospital, Beijing 100191, China

**Keywords:** infrapatellar fat pad, osteoarthritis, mesenchymal stem cells, synovium, cartilage

## Abstract

Osteoarthritis (OA) is a prevalent degenerative joint disease which affects millions of patients across the globe. The infrapatellar fat pad (IPFP) harbors diverse cell types with intricate intercellular interactions. Its mesenchymal stem cells (MSCs) and extracellular vesicles (EVs) possess significant biological functions and hold promising applications in regenerative medicine. IPFP exhibits active secretory capacity, releasing adipokines including leptin and adiponectin, along with various cytokines. Furthermore, it contains a rich neural network playing a crucial role in knee pain perception and sensation. Moreover, IPFP and synovium can be considered an integrated unit, exhibiting interactions both with each other and with cartilage. In imaging applications, IPFP is gaining widespread attention as an emerging biomarker. In clinical practice, the decision to resect or preserve IPFP remains a controversial topic. This article will review the latest research regarding the mechanism of IPFP in OA, and discuss its clinical applications, providing a theoretical basis for the prevention and treatment of OA.

## 1. Introduction

Infrapatellar pad (IPFP) is the largest fat pad within the knee joint. It is located posterior to the inferior pole of the patella, anterior to the tibia, and between the menisci and the anterior aspect of the femur [1]. Anatomical studies indicate that IPFP is a structurally heterogeneous tissue. It is composed of superficial large lobules and deeper small lobules. These features allow it to better adapt to the changes in adipose tissue during knee movements [2]. Specifically, it reduces knee joint load, expands the synovial space, supports the blood supply to the patellar tendon, and distributes lubricant within the joint space [3,4]. In short, IPFP reduces the biomechanical stress applied to the joint and helps prevent injury during joint activities, indicating its importance in maintaining the full range of motion in the knee joint [5,6].

Recently, scientists have discovered that IPFP not only serves biomechanical functions in the knee joint but also exhibits activities and functions similar to an endocrine organ. IPFP is home to a rich cellular reservoir, comprising various cell types such as fibroblasts, adipocytes, macrophages, endothelial cells, dendritic cells, smooth muscle cells, lymphocytes, and mast cells [7]. Mesenchymal stem cells (MSCs) are not independent cell types, but a collective term for a class of cells with multipotent differentiation potential and immune regulatory functions. These cells collectively contribute to the maintenance of knee joint homeostasis through a complex cellular communication network. Additionally, IPFP secretes adipokines, including leptin and adiponectin, as well as pro-inflammatory cytokines such as interleukin-6 (IL-6) and tumor necrosis factor (TNF) [8,9]. It also contains numerous nerve fibers and peripheral nerve endings that release substance P [10]. Beyond the growing understanding of its endocrine properties, researchers have increasingly recognized that IPFP is not an isolated adipose tissue but actively interacts with other knee joint tissues, including cartilage and synovium [11].

Osteoarthritis (OA), a worldwide disease involving physiological changes in joint tissues, can lead to distressing outcomes for patients, including cartilage degeneration, bone remodeling, meniscal degeneration, inflammation of synovial membrane, pain, stiffness, swelling, and restricted joint function [12,13,14,15,16]. OA is one of the main causes of disability and the seventh leading cause of disability worldwide among people over 70 years old [17]. In 2020, approximately 595 million people globally were affected by OA, representing 7.6% of the world’s population. Among individuals aged 30 years and older, the prevalence was as high as 14.8%. By 2050, the number of people living with OA worldwide is projected to approach 1 billion [18]. OA pain does not always correspond to the degree of structural damage and is influenced by multiple factors. Comorbidities such as depression, cardiovascular diseases, and sleep disorders can significantly exacerbate the pain and disability of OA patients. Age, gender, BMI, allergic diseases and other factors are considered to be related to OA [17,19,20]. As the knee is the most commonly affected joint, knee osteoarthritis (KOA) has become a significant public health challenge for healthcare systems across the globe [21].

IPFP may serve as a functional unit in the pathogenesis of OA and associated pain. IPFP plays a unique structural-mechanical role within the knee joint. Compared to the suprapatellar fat pad in OA, the IPFP exhibits distinctly different and stiffer mechanical properties, suggesting it may assume a specialized, more load-bearing structural role within the osteoarthritic knee [22]. During the progression of OA, IPFP undergoes significant pathological remodeling, including fibrosis, inflammatory cell infiltration, increased angiogenesis, and adipocyte atrophy [3,23,24]. Studies indicate that IPFP can mediate its own fibrosis, cartilage destruction, and knee joint inflammation by secreting various inflammatory factors, cytokines, and extracellular vesicles [25,26,27]. Although the mechanisms of IPFP in OA are not yet fully understood, it represents a potential therapeutic target and biomarker, offering promising avenues for the treatment and prevention of OA [23].

In this narrative review, we will explore the cellular heterogeneity and secretory profile of IPFP, and elucidate the intrinsic connections and interactions between IPFP, synovium, and cartilage. From a therapeutic perspective, we will analyze the potential applications of IPFP-derived MSCs and their extracellular vesicles (EVs), as well as the diagnostic utility of IPFP in conjunction with advanced imaging techniques. Finally, we will examine the considerations and implications regarding the surgical resection of IPFP in clinical management.

## 2. Characteristics of IPFP

### 2.1. Cell Types in IPFP

IPFP harbors a diverse cellular repertoire. The interplay between the cells—particularly the dysregulated crosstalk that drives a chronic, low-grade inflammatory state—is considered a key mechanism impacting OA progression [28]. In current studies, its cellular heterogeneity has been progressively revealed through single-cell RNA sequencing (scRNA-seq) and single-nucleus RNA sequencing (snRNA-seq). During OA, IPFP secretes inflammatory cytokines and undergoes fibrosis. IPFP under osteoarthritic conditions demonstrates significantly enhanced lymphocyte infiltration and increased vascularization. Concurrently, the gene expression levels of the pro-inflammatory cytokine IL-6 and the pro-angiogenic factor vascular endothelial growth factor (VEGF) are significantly upregulated. In addition, gene expression related to adipose tissue fibrosis and extracellular matrix (ECM) stability is altered. ECM undergoes remodeling, characterized by lower gene and protein expression of both type I collagen (COL I) and type III collagen (COL III) [29].

Research has revealed that based on canonical markers, eight cell types were identified within IPFP: fibroblasts, adipocytes, macrophages, endothelial cells, dendritic cells, smooth muscle cells, lymphocytes, and mast cells. Among these, fibroblasts were the most abundant (44.35%). The cell types can be broadly classified into mesenchymal, immune, and vascular lineages. The mesenchymal lineage includes DPP4-positive mesenchymal progenitor cells (MPCs), CD142-positive preadipocytes (preADs), mature adipocytes (ADs), biglycan-positive intermediate fibroblasts, and synovial lining layer fibroblasts. In OA, the relative amounts of these cells shift: preADs decrease from 21.9% to 7.0% of mesenchymal cells, while intermediate fibroblasts increase from 14.9% to 22.1%. Among immune cells, myeloid lineage cells such as resident macrophages and inflammatory macrophages are predominant, with inflammatory macrophages significantly expanding in OA from 32.5% to 57.5% of myeloid cells. Other identified cell types include endothelial cells (further subdivided into capillary, venous, and lymphatic subsets), mural cells (pericytes and smooth muscle cells), lymphoid cells (T cells, NKT cells, naive and memory B cells), and osteoclasts. These changes highlight the IPFP’s cellular heterogeneity and its dynamic reorganization in disease, particularly through the enrichment of profibrotic and pro-inflammatory subsets that contribute to OA progression [30].

### 2.2. Cell Interplay in IPFP

**Figure 1:**
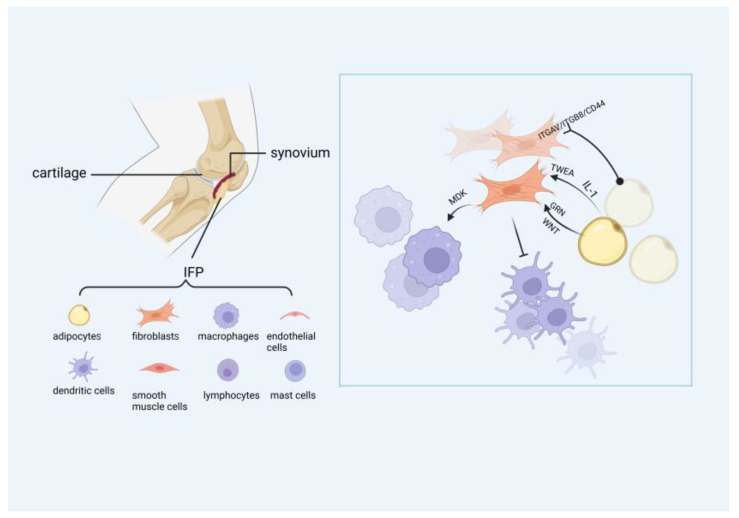
The main cell types and relationships contained in IPFP. The eight types of cells include fibroblasts, adipocytes, macrophages, endothelial cells, dendritic cells, smooth muscle cells, lymphocytes, and mast cells. Fibroblasts are the most abundant and interact with adipocytes and immune cells.

During the progression of OA, the interactions among fibroblasts, adipocytes, and immune cells play a significant role (Figure 1). In these cellular communications, fibroblasts predominantly express ligands, while macrophages, adipocytes express corresponding receptors.

Ligand-receptor pairs derived from adipocytes account for the highest proportion, and they notably interact with cluster of differentiation 44 (CD44) and integrin receptors (integrin subunit alpha V) ITGAV/(integrin subunit beta 8) ITGB8 on fibroblasts. Furthermore, interstitial inflammatory fibroblasts can enhance communication with adipocytes through the TNF-like weak inducer of apoptosis (TWEAK), IL-1, granulin (GRN), and non-canonical wingless-type MMTV integration site family (ncWNT) signaling pathways. Specifically, TWEAK inhibits lipogenesis, while activation of the GRN receptor sortilin 1 (SORT1) also suppresses lipid production. Additionally, WNT5B derived from these fibroblasts acts on frizzled class receptor 4 (FZD4) and FZD8 receptors to influence ncWNT signaling, and the activation of FZD receptors typically suppresses the activation of WNT target genes that promote adipogenesis [31]. However, WNT5B has also been shown to promote adipogenesis. This paradoxical role underscores the complexity of signaling pathways studied in OA and highlights the need for more precise mapping of OA molecular mechanisms in future research.

Fibroblasts also play an active regulatory role in modulating immune cell functions. Midkine (MDK) secreted by interstitial inflammatory fibroblasts regulates this process through multiple mechanisms. First, it directly interacts with integrin subunit beta 2 (ITGB2) via low-density lipoprotein receptor-related protein 1 (LRP1), inducing chemokine expression and thereby promoting the recruitment of neutrophils and macrophages to inflammatory sites. Second, it inhibits the development of tolerogenic dendritic cells, consequently impeding the differentiation of regulatory T cells [31].

### 2.3. IPFP-Derived MSCs

MSCs residing within IPFP constitute a functionally heterogeneous population. Current evidence suggests that their defining properties, such as self-renewal and multipotency, are primarily attributable to a distinct, rapidly self-renewing subpopulation, while other co-isolated stromal cell types lack these core stem cell characteristics [32]. MSCs, particularly research on their EVs, represent a current hotspot in regenerative medicine [33]. A comprehensive understanding of exosome mechanisms will greatly facilitate future treatments for OA. The following analyzes the mechanistic roles, potential applications and limitations of MSCs and their EVs.

As an important cellular subset within IPFP, MSCs have been the subject of various mechanistic studies to date. Studies have shown that MSCs derived from IPFP (IPFP-MSCs) can modulate the functions of synovial cells and macrophages under inflammatory conditions in vitro through their EVs. The specific mechanism involves altering inflammation-related molecular profiles and reducing the secretion of pro-inflammatory molecules. In a rat model of acute synovial/IPFP inflammation, treatment with IPFP-MSC-derived extracellular vesicles (IPFP-MSC EVs) induced robust polarization of macrophages toward the anti-inflammatory M2 phenotype within the synovial/IPFP tissue [34]. This finding highlights the critical role of MSCs in promoting anti-inflammatory responses in tissues. Furthermore, MSCs obtained from sources with varying degrees of inflammation maintain stable and similar transcriptomic profiles, with no detectable differences [35]. This suggests that MSCs possess high functional stability in tissue repair, supporting their potential use in regenerative medicine strategies. In addition, Research has revealed that IPFP-MSCs possess robust immunomodulatory activity, capable of inducing significant phenotypic shifts in cells and effectively promoting cartilage repair [36]. Moreover, studies have revealed that transforming growth factor-β (TGF-β) activates WNT/β-catenin signaling through mothers against decapentaplegic homolog 3 (SMAD3), thereby increasing the propensity for hypertrophy in IPFP-MSCs [37]. However, undesirable hypertrophic maturation of MSCs impedes their clinical application in cartilage repair. To address this limitation, researchers targeted WNT signaling: during the differentiation phase, they inhibited TGF-β-induced WNT signaling using XAV939, whereas during the expansion phase, they activated WNT signaling with CHIR99021. These results demonstrate that in-depth investigation into the regulatory mechanisms of IPFP-MSCs can provide critical support and insight for their clinical translation in regenerative medicine.

The application of IPFP-MSCs has emerged as a research focus in regenerative medicine for treating inflammatory diseases. Studies have found that compared to bone marrow-derived mesenchymal stem cells (BM-MSC) and subcutaneous adipose tissue-derived MSCs, IPFP-MSCs demonstrate superior chondrogenic potential [36,38]. Studies have demonstrated that co-culture of IPFP-MSCs with chondrocytes enhances the expression of chondrogenic genes [39]. This finding provides a valuable reference for the preconditioning and clinical utilization of IPFP-MSCs. To effectively evaluate patient drug responses in the context of OA, a study utilized a three-dimensional organ-on-a-chip approach to direct MSCs toward the chondrogenic lineage. Through targeted gene expression analysis and morphological assessment, it was demonstrated that combining MSCs with the organ-on-a-chip platform could provide a viable alternative to cartilage biopsy [40]. This research may pave the way for personalized therapeutic strategies in OA and enable an in-depth exploration of individual genetic susceptibility to OA. Additionally, in a study on meniscal regeneration, IPFP-MSCs were loaded into a bioink and fabricated via 3D bioprinting using a silk fibroin-extracellular matrix-gelatin (SF-ECM-G) hydrogel [41]. This approach provides a novel regenerative solution for meniscal repair.

**Figure 2:**
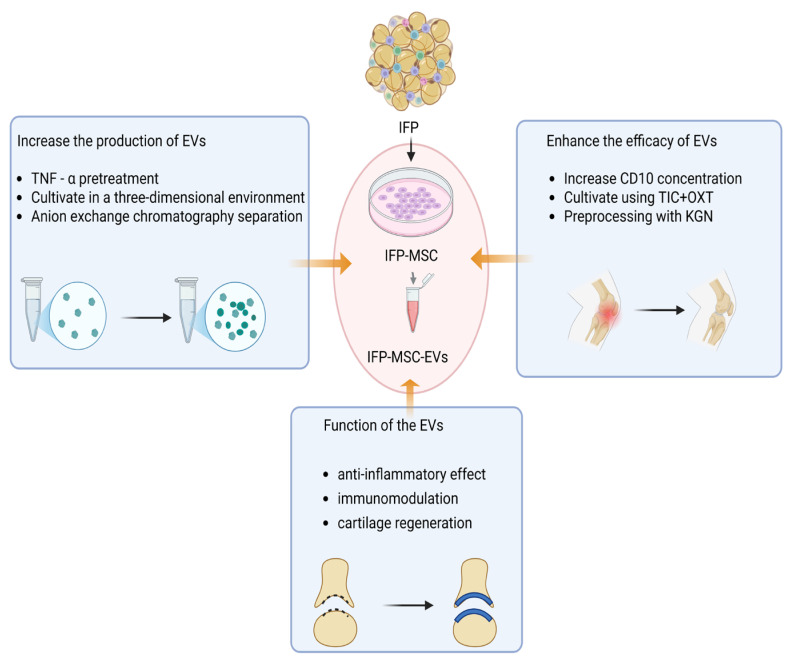
Strategies for promoting the production and enhancing the efficacy of IPFP-MSC-derived EVs. The EVs exert multiple functions including anti-inflammatory activity, immune regulation, and cartilage repair. The production of EVs was improved by TNF-α pretreatment, three-dimensional culture, and separation via anion exchange chromatography. The efficacy was enhanced by increasing CD10 concentration, cultivation with TIC + OXT, and pretreatment with KGN.

EVs are enriched with diverse RNA species and are considered the primary mediators through which IPFP-MSCs exert their therapeutic effects (Figure 2) [42]. Recently, research on IPFP-MSC-derived EVs has gained significant momentum, driven by the aim to develop a cell-free therapy that avoids the potential side effects associated with the direct intra-articular injection of mesenchymal stem cells [43]. Notably, one study found that EVs derived from IPFP-MSCs accelerated tendon-bone healing and intra-articular graft remodeling after anterior cruciate ligament reconstruction (ACLR). The underlying mechanism for this therapeutic effect may involve immunomodulation through macrophage polarization, thereby expanding the potential application scenarios of IPFP-MSC-derived EVs [44].

Substantial research efforts are dedicated to improving the yield of EVs. Regarding secretion capacity, TNF-α preconditioning has been shown to enhance exosome secretion by IPFP-MSCs. The underlying mechanism involves TNF-α preconditioning activating the phosphatidylinositol 3-Kinase/protein kinase B (PI3K/AKT) signaling pathway in IPFP-MSCs [45]. In addition, A study utilized a vertical wheel bioreactor with microcarrier suspension culture to grow primary IPFP-MSCs in a three-dimensional environment for enhanced extracellular vesicle (EVs) release. This approach yielded a greater quantity of EVs compared to conventional two-dimensional culture methods [46]. EVs derived from IPFP-MSCs have been successfully isolated via anion-exchange chromatography and demonstrated efficacy in suppressing OA progression in a mouse model, providing a promising direction for future clinical translation [47].

In the pursuit of enhancing the functional efficacy of EVs, various hormones and signaling molecules have been explored for exosome pretreatment, aiming to extend their current application scope beyond the limitations of OA. One study isolated small extracellular vesicles (sEVs) from IPFP-MSCs cultured based on CD10 High and CD10 Low expression levels and evaluated their sEV miRNA cargo. The CD10 High sEVs group demonstrated effective immunomodulatory and chondroprotective properties, significantly influencing the functions of synovial cells and chondrocytes under inflammatory conditions in vitro [48]. Moreover, IPFP-MSCs cultured with inflammatory/fibrotic cocktail (TIC) with oxytocin (OXT) yielded EVs with substantially enhanced immunomodulatory and chondroprotective potential. Mechanistically, these engineered EVs promoted M2 macrophage polarization, suppressed M1 pro-inflammatory markers, and upregulated key cartilage-related genes fibromodulin (FMOD), tissue inhibitor of metalloproteinase 1(TIMP1), and C-X-C Motif Chemokine Ligand 8 (CXCL8)), thereby enhancing their role in ECM remodeling and joint homeostasis [49]. Additionally, preconditioning IPFP-MSCs with Kojibiose (KGN) yields EVs with an enhanced chondrogenic capacity. These EVs effectively promote chondrocyte proliferation and the expression of cartilage-specific genes and proteins. Furthermore, KGN-preconditioned EVs demonstrate superior efficacy in promoting the repair of articular cartilage defects [50]. In addition, researchers have engineered CD10-bound sEVs from IPFP-MSCs to target calcitonin gene-related peptide (CGRP) while preserving their anti-inflammatory phenotype. In this approach, human IPFP-MSC cultures were transduced with an adeno-associated virus (AAV) vector carrying a green fluorescent protein (GFP)-labeled gene encoding a calcitonin CGRP antagonist peptide. This innovative strategy effectively addressed the inflammatory effects mediated by the regulatory neuropeptides substance P and CGRP, resulting in significant anti-inflammatory and analgesic effects [51].

However, there are still key limitations that may affect the translational applicability of IPFP-derived MSCs and their EVs. First, stem cells within OA IPFP may undergo functional remodeling in the chronic inflammatory microenvironment. Studies have shown that OA-IPFP-derived stem cells exhibit elevated expression of immune activation markers such as human leukocyte antigen–DR isotype (HLA-DR) and Fas receptor/Fas ligand (Fas/FasL), while demonstrating low expression of cluster of differentiation 38/Nicotinamide adenine dinucleotide glycohydrolase (CD38/NADase). This altered expression profile may compromise their ability to effectively counteract inflammatory responses within the joint [52]. In addition, the inter-donor heterogeneity of IPFP-MSCs constitutes another key limitation. Research has demonstrated that the degree of inflammation within the OA-IPFP itself varies significantly among patients. [53] Consequently, MSCs isolated from this source are likely to exhibit considerable functional heterogeneity, which is also dependent on the disease stage.

### 2.4. Key Bioactive Secretions of IPFP

IPFP serves as a critical site for the secretion of inflammatory mediators within the knee joint, releasing adipokines such as leptin, adiponectin, resistin, as well as cytokines including IL-6 and IL-8, while simultaneously being regulated by these factors [54]. Leptin, adiponectin, and resistin all demonstrate a certain positive correlation with the risk of knee OA [55,56]. However, Mendelian randomization studies have indicated that leptin played a significant role in knee OA, while the effects of adiponectin and resistin are relatively weak [57]. In addition, experimental transplantation of adipose tissue from wild-type mice into lipodystrophic (LD) recipients reconstituted systemic leptin levels and triggered OA pathology, demonstrating a causal role for adipocyte-derived leptin in OA pathogenesis [58].Thus, this review will primarily focus on the mechanisms of leptin in OA and its potential therapeutic approaches.

IPFP, local source of leptin, secretes this adipokine which acts directly on joint tissues and thereby influences the progression of OA [55]. Previous research has elucidated the mechanisms of leptin in OA. Leptin contributes to OA pathogenesis by activating multiple signaling pathways, including Janus Kinase/signal transducer and activator of transcription (JAK/STAT), mitogen-activated protein kinase (MAPK), and PI3K/AKT, ultimately leading to cartilage degradation and cellular senescence. Furthermore, it promotes the secretion of inflammatory factors such as IL-6 and IL-8 by synovial fibroblasts, thereby exacerbating synovitis [59,60].

Recently, more studies have provided new insight into Leptin’s role in the progression of OA. One Study, employing methodologies including fat pad implantation and multi-omics analyses, demonstrated that leptin derived from IPFP upregulates the expression of complement factor D (FD), subsequently promoting structural joint damage. Notably, elevated FD expression is associated with the suppression of nociceptive signaling and a reduction in pain sensitivity [55]. This divergence between structural OA progression and pain response provides profound molecular insight into OA as a systemic disorder. Additionally, studies have revealed that the leptin–leptin receptor signaling pathway activates the STAT3 transcription factor, thereby upregulating the expression of fibroblast growth factor 7 (FGF7). This in turn drives the senescence of skeletal stem cells (SSCs), leading to abnormal subchondral bone remodeling and thereby exacerbating OA progression [61].Beyond the direct role of leptin in OA, cells expressing the leptin receptor (LEPR) can independently promote OA progression by driving fibrotic processes [62]. Mechanistic studies reveal that upregulation of adhesion G protein-coupled receptor F5 (ADGRF5) signaling in leptin receptor^+^ (LEPR^+^) cells enhances cellular proliferation, suppresses differentiation, and ultimately leads to fibrosis [62]. This research demonstrates that the pathophysiological effects of leptin may not be solely mediated indirectly via adipocyte-derived leptin secretion, but can also be directly orchestrated through the activation of LEPR-expressing cells within the adipose pad, such as stromal vascular fraction (SVF) cells.

As a pivotal adipokine in OA, leptin has emerged as a promising therapeutic target, with several targeted strategies demonstrating potential for clinical intervention. First, regarding the previously discussed LEPR^+^ cells, therapeutic approaches targeting ADGRF5 and its downstream genes show considerable promise. Furthermore, in obesity-associated OA, metformin indirectly mitigates leptin-induced damage to chondrocytes and macrophages by reducing leptin secretion from adipose tissue. This finding expands the known mechanisms of metformin beyond direct cartilage protection and synovial immunomodulation, advancing its clinical translation for OA treatment [63]. Additionally, mammalian target of rapamycin (mTOR) inhibitors and leptin receptor antagonists are projected to offer significant therapeutic benefits [55,64]. From a biomarker perspective, measuring leptin levels in serum or synovial fluid may facilitate early OA diagnosis, prognosis assessment, and treatment monitoring [60].

### 2.5. The Neural Network Within IPFP

IPFP is densely innervated by a neural network, rendering it highly sensitive to pain [5,65,66]. Furthermore, this rich neural network within IPFP contributes not only to pain-related homeostasis of the knee joint, but also plays a role in the inflammatory processes of OA. Understanding the interactions between IPFP and nerves will facilitate the development of nerve-targeting therapies, offering potential therapeutic strategies for conditions such as anterior knee pain syndrome and OA.

Regarding the neural origins, the nerves in IPFP are primarily derived from a branch of the tibial nerve arising from the anterior fibers in the popliteal fossa [24]. Additional neural contributions come from the femoral nerve, common peroneal nerve, and saphenous nerve [67]. From a spatial distribution perspective, the neural network within IPFP exhibits connections with the synovial nerves, although distinct distribution patterns exist between them [68]. Furthermore, a certain proportion of the sensory fibers in IPFP are nociceptive fibers. For example, IPFP is notably rich in type IVa nociceptive nerve endings. This evidence indicates that the nerves within IPFP are involved in the complex pain regulatory network of the knee joint.

The neural-mediated pain and inflammatory mechanisms in IPFP interact, forming a closed-loop cycle that exacerbates knee joint inflammation. Sensory fibers release neuropeptides such as Substance P and CGRP positioning IPFP as a key source of anterior knee pain and inflammation [65]. SP initiates and significantly increases the production of pro-inflammatory cytokines (e.g., IL-1β, IL-6, TNF-α), promotes macrophage chemotaxis, heightens sensitivity to noxious signals, and leads to vasodilation, plasma protein extravasation, and tissue edema [5,65,69]. CGRP induces pain signaling and promotes endothelial cell proliferation and angiogenesis (neovascularization) [65]. Concurrently, these inflammatory responses further stimulate nerves in a reciprocal manner, creating a vicious cycle that worsens knee joint homeostasis.

Liebmann et al. engineered IPFP-derived MSCs via adeno-associated virus (AAV)-mediated transduction of a CGRP antagonist. The resulting sEVs block CGRP-mediated pain signaling and deliver anti-inflammatory, analgesic, and reparative cargo. They polarize M1 macrophages to the M2 phenotype and modulate neuronal pain pathways, achieving dual anti-inflammatory and analgesic effects [51]. Future research should focus on developing drugs targeting key neuropeptides such as SP and CGRP. A deeper investigation into the neuro-pain–inflammatory mechanisms within IPFP will provide further therapeutic possibilities. This dual approach, combining advanced vesicle-based delivery systems with targeted molecular strategies, holds significant promise for creating more effective treatments for IPFP-related pathologies.

## 3. IPFP and Its Interplay with Synovium and Chondrocyte in the Context of OA

### 3.1. IPFP and Synovium as a Functional Unit

Being an intra-articular adipose tissue, IPFP is in direct anatomical continuity with synovial and cartilaginous structures, underscoring its relevance to joint homeostasis. Studies have indicated a close relationship between IPFP and synovium, demonstrating that DPP4^+^ mesenchymal progenitor cells located in the synovial sub-lining layer possess bidirectional differentiation potential [30]. These mesenchymal cells can differentiate into both preadipocytes in IPFP and synovial lining fibroblasts. Moreover, IPFP-derived fibroblastic cells secrete netrin-4 (NTN4) and contribute to cartilage degradation during the progression of OA [25]. It is noteworthy that NTN4 is highly expressed in synovial lining fibroblasts, suggesting a strong link between IPFP inflammation and synovial involvement [70]. Furthermore, flow cytometric analysis of inflammatory cells within IPFP and synovium has revealed similarities in their immune cell composition [71]. Additionally, IPFP secretes adipokines such as adiponectin and leptin into the synovial fluid, illustrating regulations between IPFP and synovium [2]. Prostaglandin E2 (PGE2), a key mediator in the interaction between IPFP and synovium, promotes inflammatory responses in fibroblast-like synoviocytes [72]. To sum up, these findings collectively suggest that IPFP and synovium may function as a coordinated unit in the pathogenesis of OA and the maintenance of knee joint homeostasis.

### 3.2. The Impact of IPFP and Synovium on Cartilage

As a vulnerable site in OA, cartilage also exhibits strong cellular and molecular connections with IPFP and synovium. Within OA progression, both IPFP and synovium pose impacts on cartilage homeostasis, and these influences display both similarities and differences.

Studies have indicated that IPFP plays a significant role in chondrocyte degradation as well as cellular aging within chondrocytes. EVs derived from IPFP, carrying abundant let-7b-5p and let-7c-5p, exert detrimental effects on cartilage homeostasis by downregulating the senescence-negative regulator LBR (lamin B receptor). In addition, IPFP-derived osteopontin (OPN) plays a crucial role in OA progression, promoting chondrocyte hypertrophy as well as IPFP fibrosis [73]. Furthermore, osteoarthritic IPFP promotes cartilage degradation and inflammation by activating the p38 Mitogen-Activated Protein Kinase (p38MAPK) and extracellular signal-regulated kinase 1/2 (ERK1/2 pathways). It is worth mentioning that IL-1β and TNF-α act as key factors in this mechanism, whereas the common pro-inflammatory cytokine IL-6 does not appear to be involved [74]. This highlights the complexity of the biomolecular mechanisms within IPFP and surrounding structures, suggesting that the development of targeted therapies should be based on a thorough understanding of the underlying molecular pathways. Beyond specific mechanisms unique to IPFP, both IPFP and synovium influence chondrocytes in similar yet distinct ways. Intermediate fibroblasts and macrophages in both IPFP and the synovium exhibit enhanced expression of apolipoprotein E (APOE), which stimulates chondrocyte degradation through mitogen-activated protein kinase/extracellular signal-regulated kinase (MAPK/ERK) and nuclear factor-κB (NF-κB) signaling pathways [30]. However, which pathway is primarily responsible for the deterioration remains unclear, but identifying the precise pathway in future studies would greatly benefit the design of tailored treatments for OA.

Moreover, research has revealed the roles of macrophages in IPFP and synovium in impairing cartilage health and demonstrated differences in the immune cell expression profiles between these two tissues [75]. The exact mechanism of action of IPFP-derived macrophages has not been fully elucidated, but it is reported that macrophage numbers increase during OA [76]. IPFP macrophages inhibit chondrogenesis of mesenchymal stem cells, thereby compromising cartilage integrity. On the other hand, the mechanism by which synovial macrophages damage cartilage is relatively well-defined: synovial macrophages secrete pro-inflammatory signaling molecules such as alarmins and cytokines including IL-1 and tumor necrosis factor (TNF)-α. In addition, bioactive factors secreted by CD14^+^ synovial macrophages activate the production of matrix metalloproteinases in synovial fibroblasts. [77]. Unlike immune cells derived from IPFP, which impede chondrocyte regeneration, substances secreted by synovial-derived immune cells stimulate chondrocytes to produce more ECM-degrading enzymes and further promote cartilage matrix destruction [75].

In addition to their detrimental effects, IPFP and synovium also exert certain protective and reparative functions on chondrocytes in OA. Fibroblast growth factor 21 (FGF21) inhibits chondrocyte senescence and ECM degradation in OA through the SIRT1-mTOR signaling pathway [78]. This effect stands in contrast to the previously discussed cartilage-damaging role of macrophages, further underscoring the cellular heterogeneity within IPFP and IPFP-synovium integrated unit [75].

### 3.3. The Effect of Cartilage on IPFP and Synovium

**Figure 3:**
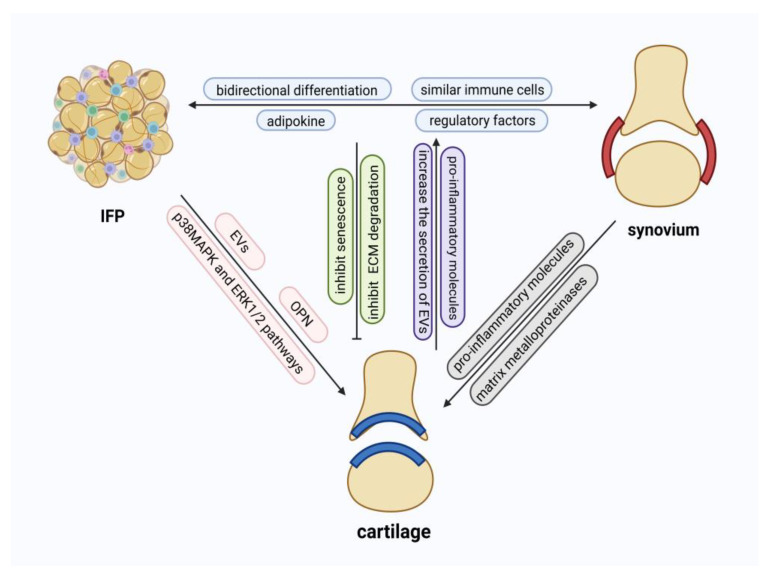
Functional crosstalk between IPFP, synovium, and cartilage. IPFP and synovium exhibit bidirectional differentiation, similar immune cell profiles, and shared adipokine regulatory factors, acting together as an integrated functional unit. IPFP and synovium reciprocally interact with cartilage: IPFP and synovium inhibit senescence and ECM degradation in cartilage, whereas cartilage promotes pro-inflammatory molecule secretion and EVs release from IPFP and synovium. Furthermore, IPFP regulates cartilage through EVs, OPN, and the p38 MAPK and ERK1/2 signaling pathways, while synovium modulates cartilage via pro-inflammatory molecules and matrix metalloproteinases.

Beyond the impact of IPFP-synovium integrated unit on cartilage, senescent chondrocytes can also exert reciprocal effects on cells within IPFP and synovium. Studies have shown that senescent chondrocytes release pro-inflammatory molecules into neighboring cells and tissues, stimulating osteocytes and synovial fibroblasts and impacting their already limited regenerative potential. Moreover, senescent chondrocytes increase the secretion of EVs, which promote intercellular communication in bystander fibroblasts and drive the aging of nearby tissues [78]. However, in-depth investigations into the specific mechanisms involving IPFP in this context remain relatively limited. In summary, knee joint aging involves complex inter-tissue interactions, including but not limited to communication among cartilage, synovium, and IPFP. These tissues may engage in feedback regulation that collectively promotes the progression of OA. This insight suggests that IPFP, synovium, and cartilage can be analyzed and explored as an integrated unit in the context of OA and aging-related mechanisms (Figure 3).

## 4. Imaging Applications of IPFP

Early diagnosis plays a crucial role in the prevention and treatment of OA. IPFP, as an important intra-articular adipose tissue in the knee, has been recognized to play a key role in the inflammatory, metabolic, and structural changes in KOA, and is considered a potential biomarker for predicting OA progression. Conventional semi-quantitative assessment methods are limited by suboptimal accuracy, high subjectivity, and poor reproducibility. Recent advances in biomarker and imaging research indicate that the morphological and signal characteristics of IPFP, combined with emerging imaging technologies and predictive modeling approaches, are providing new avenues for the early diagnosis of OA [79].

First, a growing body of evidence indicates that the morphological characteristics of IPFP are closely associated with the progression of KOA, highlighting its potential as a novel biomarker [80]. Longitudinal studies utilizing the osteoarthritis Initiative (OAI) cohort data have demonstrated the significance of dynamic changes in IPFP, revealing that longitudinal increases in IPFP size, rather than baseline measurements, serve as a strong predictor of KOA progression [81]. This underscores the clinical importance of monitoring adipose tissue dynamics. IPFP volumetric parameters, particularly global volume and low-signal intensity volume, have been identified as sensitive indicators for predicting long-term structural deterioration and the risk of knee replacement (KR) in KOA. Notably, a smaller IPFP volume exhibits a significant inverse association with the risk of KR, potentially indicative of a protective mechanism [80]. Additionally, the signal characteristics of IPFP also demonstrate considerable potential as biomarkers. Utilizing DIXON technology, quantitative assessment of proton density fat fraction (FF) and T2* relaxation times can effectively detect pathological alterations in IPFP of patients with KOA. A decrease in FF indicates reduced fat content resulting from IPFP edema, inflammation, and fibrosis, while a shortened T2* value may be associated with increased water content and local magnetic field inhomogeneity [82]. This technique offers the advantages of being non-invasive, rapid, and reproducible, indicating that FF and T2* hold promise as novel imaging biomarkers for KOA assessment. Additionally, studies have found that quantitative IPFP signal intensity metrics may exhibit a strong association with radiographic information, revealing a link between short-term alterations in IPFP signal and long-term osteoarthritis outcomes [83]. Interestingly, however, research indicates no significant correlation between IPFP signal characteristics and pain progression, providing direction for future investigations integrating multi-center data and pathological mechanisms. These studies provide new insights into the role of IPFP in KOA pathogenesis and hold significant potential for clinical translation.

Recently, numerous advanced technologies and novel information processing methods have emerged in the assessment of IPFP. These technological and data-analytic approaches are poised to enhance the biomarker potential of IPFP, thereby facilitating effective prevention and treatment strategies for KOA. Specifically, an MRI-based nomogram (OA-ASN), which integrates quantitative parameters of IPFP, T1rho, and T2 mapping, has been developed to achieve precise risk stratification for early-stage OA. This integrative tool holds significant promise for improving early diagnosis and advancing personalized medicine [84]. A multi-task deep learning system (DLS) has been developed and validated to automatically classify nine common knee abnormalities, including IPFP lesions from knee MRI scans. This advancement underscores the substantial potential of artificial intelligence in musculoskeletal imaging [85]. In addition, research utilizing ultrasound elastography has investigated the relationship between the elastic properties of IPFP and anterior knee pain in KOA patients, offering new perspectives for the clinical diagnosis of painful IPFP conditions [86]. MRI-based three-dimensional texture analysis offers an objective and quantitative method for IPFP assessment. It involves segmenting the IPFP on fat-suppressed MRI scans, extracting 20 reliable voxel-based texture features (e.g., gray-level histogram, gray-level co-occurrence matrix, and run-Length matrix indices; ICC > 0.75) to quantify subtle signal heterogeneity, and integrating them into a unified IPFP texture score via LASSO regression [87]. Furthermore, the application of histogram analysis to IPFP FF evaluation has uncovered the potential of fat distribution heterogeneity as an indicator of KOA severity, laying the groundwork for developing quantitative imaging tools [88].

## 5. The Clinical Controversy over IPFP Resection

In total knee arthroplasty, IPFP frequently obstructs the surgical field of view, prompting most surgeons to opt for partial resection to enhance exposure of the femoral and tibial insertions of the anterior cruciate ligament (ACL). However, IPFP should be recognized as a multifunctional organ in which its pathological roles are intricately intertwined with its physiological functions. When considering any therapeutic interventions, such as resection, its dual impacts on inflammation and pain perception versus biomechanical support must be carefully weighed [89]. The evidence synthesized in this review indicates that the impact of IPFP preservation is not uniform but appears to vary based on individual patient factors, such as disease stage and inflammatory status.

First, literature indicates that under certain conditions, resection of IPFP may be beneficial, such as by reducing inflammation, improving surgical exposure, or optimizing the OA process. An animal study involving guinea pigs demonstrated that IPFP resection decreased OA-related inflammatory mediators, including IL-6 and MCP-1, and enhanced cartilage structure and biomechanical performance [90]. This finding suggests that IPFP resection may attenuate OA progression through anti-inflammatory mechanisms, implying that resection in early-stage OA could facilitate recovery. Moreover, a clinical study noted that excision of pathologically altered IPFP, such as fibrotic or inflammatory tissue, improved postoperative knee function [91]. Based on long-term follow-up data, arthroscopic IPFP resection effectively reduces anterior knee pain and improves functional scores, with most patients returning to prior activity levels. Importantly, it demonstrates a favorable long-term safety profile, without radiographic evidence of patellofemoral osteoarthritis progression or patellar height alteration [92]. Based on follow-up data, arthroscopic partial IPFP excision significantly reduces pain and improves functional scores in KOA patients, while also promoting cartilage and joint structure health. The procedure demonstrated a favorable safety profile within the study period [93]. In summary, it is observed that these scenarios favoring resection likely arise when IPFP has undergone significant pathological changes or is subjected to high biomechanical stress.

In addition, studies support the preservation of IPFP to optimize postoperative function. IPFP preservation may offer slight benefits for early knee functional recovery, particularly in the context of total knee arthroplasty (TKA) [94]. This minor advantage could potentially enhance patient satisfaction to some extent. Based on systematic review and meta-analysis, IPFP preservation during TKA significantly reduces early postoperative complications and short-term anterior knee pain compared to complete resection. Importantly, while resection is associated with patellar tendon shortening and a slight decrease in knee flexion at 12 months, it does not impact long-term pain or functional outcomes [95]. Moreover, a systematic review and meta-analysis indicated that the preservation group had a lower incidence of anterior knee pain during short-term follow-up compared to the resection group, suggesting that IPFP preservation might be preferable over resection for patients undergoing primary TKA [96]. In summary, preserving IPFP based on specific circumstances in surgeries focused on functional recovery and pain reduction could play a significant role in vascular and biomechanical protection, even if this protective effect is not always pronounced.

Furthermore, literature indicates that resection and preservation of IPFP show no statistically significant differences in primary outcomes such as pain, function, and complications, allowing surgeons to opt for resection to meet intraoperative needs. A randomized controlled trial (RCT) evaluating the impact of IPFP resection on function, pain, and ultrasonographic appearance of the patellar tendon found no intergroup differences, with ultrasound confirming similar patellar tendon parameters [97]. A systematic review assessed the effects of IPFP preservation versus resection on postoperative outcomes, including range of motion, patellar height, patient-reported scores, and anterior knee pain. It concluded that preservation status did not influence the results [98]. Additionally, a double-blind RCT demonstrated no disparities in gait parameters, KOOS scores, or pain between resection and preservation groups [99]. These studies collectively suggest that IPFP resection does not significantly affect knee joint homeostasis in surgical contexts. Thus, when IPFP substantially obstructs the surgical view, surgeons may resect it to ensure procedural efficacy.

In summary, the reviewed evidence suggests that the management of the IPFP, whether to resect or preserve during TKA and ACLR is not uniform. Preservation of a normal IPFP that does not compromise exposure may offer functional advantages, highlighting the need for individualized evaluation based on intraoperative findings. Conversely, if IPFP exhibits pathological changes or obstructs exposure, resection can be performed safely and may contribute to inflammation reduction. Clinical decision-making ought to carefully weigh surgical requirements, patient-specific factors, and the status of IPFP, avoiding uniform application (Table 1).

## 6. Conclusions

With the continuous exploration of the endocrine mechanisms in IPFP, it has become an important research target for knee joint homeostasis. At present, at the cellular and molecular level, although the application of single-cell and spatial multi-omics technologies has deeply analyzed the microenvironment of cells in IPFP, there is still room for research regarding its complete cell atlas, cell-to-cell interactions, and cellular heterogeneity. In the future, research can start from IPFP-synovium integrated unit and the pathways of exosome action, to deeply analyze its cellular environment and identify more precise and effective therapeutic targets. Additionally, in clinical applications, as a new generation biomarker, the imaging-based quantitative assessment criteria for various parameters of IPFP are still insufficient. In the future, the value of IPFP as an OA biomarker can be enhanced through data collection and optimization of imaging techniques. Moreover, standardized evaluation of IPFP can also help clinicians decide whether to remove it during surgery based on the condition of patients.

## Figures and Tables

**Table 1:** The recent progress on preserving or removing IPFP.

Year	Research Subject	Research Results	Research Conclusion	Ref.
2025	100 patients with end-stage KOA underwent TKA	Removing IPFP tissue can significantly improve early postoperative knee joint mobility, enhance life satisfaction, and reduce the impact on PT structure.	in favor of resection of IPFP	[91]
2025	1 study (18 patients; 18 knees)	Resecting IPFP is effective for IFPIS patients, significantly improving clinical scores with no significant long-term radiological changes.	in favor of resection of IPFP	[92]
2024	37 KOA patients who underwent partial IFP excision	Excision of the IPFP reduces pain and improves function in KOA patients, also promoting cartilage health.	in favor of resection of IPFP	[93]
2025	57 patients with primary knee inversion underwent unilateral computer-assisted knee replacement surgery	IPFP preservation during TKA reduces the incidence and severity of subacute postoperative AKP.	in favor of preservation of IPFP	[94]
2025	21 studies (3573 patients; 4107 knees)	Preserving IPFP is superior to IPFP resection, as it reduces early postoperative complications and short-term anterior knee pain, and avoids patellar tendon shortening and slight flexion decrease at 12 months	in favor of preservation of IPFP	[95]
2016	7 studies (2734 patients; 3258 knees)	Retaining IPFP may be superior to IPFP resection, as the incidence of anterior knee pain is relatively low after short-term follow-up.	in favor of preservation of IPFP	[96]
2022	65 consecutive patients undergoing TKA for OA	During TKA, IPFP resection will not affect postoperative functional outcomes, pain scores, patellar tendon length and thickness, or ultrasound structure.	IPFP can be removed	[97]
2025	12 studies (3383 patients; 2354 knees).	There is no significant difference in the incidence of anterior knee pain, the joint range of motion, and the patellar height PROMS.	IPFP can be removed	[98]
2024	85 patients who received TKA treatment for primary OA	Similar functional outcomes are achieved after TKA with or without resection of Hoffa’s fat pad.	IPFP can be removed	[99]

## Data Availability

No new data were created or analyzed in this study. Data sharing is not applicable to this article.
